# Predicting Suicide Among US Veterans Using Natural Language Processing-enriched Social and Behavioral Determinants of Health

**DOI:** 10.21203/rs.3.rs-4290732/v1

**Published:** 2024-04-23

**Authors:** Avijit Mitra, Kun Chen, Weisong Liu, Ronald C. Kessler, Hong Yu

**Affiliations:** University of Massachusetts Amherst; University of Connecticut; University of Massachusetts Lowell; Harvard Medical School; University of Massachusetts Amherst

## Abstract

Despite recognizing the critical association between social and behavioral determinants of health (SBDH) and suicide risk, SBDHs from unstructured electronic health record (EHR) notes for suicide predictive modeling remain underutilized. This study investigates the impact of SBDH, identified from both structured and unstructured data utilizing a natural language processing (NLP) system, on suicide prediction within 7, 30, 90, and 180 days of discharge. Using EHR data of 2,987,006 Veterans between October 1, 2009, and September 30, 2015, from the US Veterans Health Administration (VHA), we designed a case-control study that demonstrates that incorporating structured and NLP-extracted SBDH significantly enhances the performance of three architecturally distinct suicide predictive models - elastic-net logistic regression, random forest (RF), and multilayer perceptron. For example, RF achieved notable improvements in suicide prediction within 180 days of discharge, with an increase in the area under the receiver operating characteristic curve from 83.57–84.25% (95% CI = 0.63%–0.98%, p-val < 0.001) and the area under the precision recall curve from 57.38–59.87% (95% CI = 3.86%–4.82%, p-val < 0.001) after integrating NLP-extracted SBDH. These findings underscore the potential of NLP-extracted SBDH in enhancing suicide prediction across various prediction timeframes, offering valuable insights for healthcare practitioners and policymakers.

## Introduction

Suicide has consistently ranked among the primary causes of mortality in the US for decades, with a substantial 35.6% increase from 2000 to 2021^1^. In 2021 alone, suicide accounted for 48,183 fatalities in the US^1^, while the global toll surpassed 700,000 ^2,3^. Existing data indicates a higher suicide rate among Veterans than non-veteran adults over the last decade and notably, Veterans are experiencing a more pronounced increase of suicide risk ^4^. Prior studies found that 80% of suicide victims were in contact with their primary care providers in the year preceding their death, and within the same timeframe, 25.7–31% had sought mental health care^5,6^. This puts the healthcare providers in a unique position to contribute, and a better predictive tool may assist them in mitigating the prospective risk of suicidal events.

Social and behavioral determinants of health (SBDH) encompass factors such as socioeconomic status, access to healthy food, education, housing etc. that wield strong influence over an individual’s health outcomes ^7^. Prior studies established strong relationships between SBDHs and suicidal behaviors^8–12^. For example, social disruptions (e.g., relationship dissolution, financial insecurity, legal problems, and exposure to childhood adversity) exhibit significant associations with suicidal behaviors^8,12–15^. However, leveraging SBDHs for predicting suicide has presented challenges, primarily due to the limitations in structured data sources, such as ICD codes, for capturing comprehensive and reliable SBDH information. Unstructured clinical notes, enriched with detailed SBDH information, can play a vital role in this regard^1 2,16^.

The increasing use of Electronic Health Records (EHR) in the US has stimulated efforts to identify patients at suicide risk using EHR data. This has resulted in data mining and machine learning approaches to predict suicidal behavior and suicide mortality among patients in large healthcare systems^17,18^. While most of the existing work on suicide risk assessment using SBDH has focused on structured data sources, unstructured EHR notes represent a relatively untapped data source that can be accessed relatively inexpensively. With the advent of advanced natural language processing (NLP) techniques, there are large opportunities to automate SBDH extraction from EHR notes to augment the structured data, aiding healthcare providers with a more holistic view of a patient’s overall health status and suicide risk^19,20^.

The US Department of Veterans Affairs (VA) operates the largest integrated healthcare network in the country, with a national EHR system used by more than 1,200 medical centers and clinics^21^. With great public concern about the health of Veterans, the VA presents a unique opportunity to fully leverage its data for the exploration of suicide-related predictive modeling. In this study, we conducted the first retrospective case-control study to examine the impact of both structured and NLP-extracted (from unstructured notes) SBDH on suicide death among Veterans. We evaluated three architecturally distinct suicide prediction models across multiple prediction windows. As detailed below, our findings showed that SBDHs can improve all models’ predictive performance across different prediction windows.

## Methods

### Data Source and Study Design

In this study, we used inpatient and outpatient EHRs from the US Department of Veterans Affairs Veteran Health Administration (VHA) Corporate Data Warehouse. We included all discharges from outpatient emergency room and inpatient care between October 1, 2009 (start of Fiscal Year [FY] 2010) and September 30, 2015 (end of FY 2015) and following Kessler et al.^22^, the unit of analysis was hospital discharge. Our study protocol was approved by the institutional review board of VA Bedford Health Care. The Transparent Reporting of a Multivariable Prediction Model for Individual Prognosis or Diagnosis (TRIPOD)^23^ reporting guidelines were followed.

Cases were defined as discharges followed by deaths from suicide (according to National Death Index^24^ with International Classification of Diseases (ICD), Tenth Revision, codes X60–X84, Y87.0, and/or U03 as underlying cause of death) in the next D days (‘prediction window’). From each discharge, we established a 2-year retrospective ‘observation window’ to aggregate all relevant information for prediction. Each case was randomly matched, without replacement, to 5 discharges that were not followed by suicide in the prediction window (controls), on discharge type and date (± 1 year). Our discharge inclusion criteria include 1) at least one diagnosis or procedure record within the observation window, 2) patients at least 18 years old with no conflicting demographic information, and 3) discharges at least D days before the study end date (September 30, 2015).

We analyzed 4 prediction windows − 7, 30, 90, and 180 days, resulting in 4 cohorts. For each discharge, our task was to predict death by suicide within the prediction window, given all data from the observation window. We put aside the discharges from FY 2015 as the hold-out test data and used remaining discharges for training.

### Predictor Construction

We categorized all predictors into four groups: demographics, codes, suicide behavioral (SB) information, and SBDH. The demographic predictors contain patients’ race, gender, age, and marital status. Codes include diagnosis codes, procedure codes, medication codes. Since diagnoses and procedure codes are hierarchical, encoding all of them may lead to overfitting. We therefore used the single-level Clinical Classification Software (CCS) [1] to categorize them. This led to 283 categories for diagnosis codes and 248 categories for procedure codes. We also categorized medication codes following VA National Formulary^25^ (VANF) drug classification. More details are available in Appendix 1. SB information includes suicide attempt (SA) and ideation (SI), obtained using the phenotype algorithm available through the VA’s Centralized Interactive Phenomics Resource (CIPHER)[2].

SBDHs were identified from structured data using ICD-9 and VHA stop codes (structured SBDH), and clinical notes using NLP (NLP-extracted SBDH). Structured SBDHs included 6 factors - social or familial problems, employment or financial problems, housing instability, legal problems, violence, and non-specific psychosocial needs. NLP-extracted SBDHs were obtained from unstructured clinical notes using a transformer-based^26^ NLP system^12^, and comprised 12 factors - social isolation, job or financial insecurity, housing instability, legal problems, barriers to care, violence, transition of care, food insecurity, substance abuse, psychiatric symptoms, pain, and patient disability. SBDHs were extracted from the following 9 note types - emergency department notes, nursing assessments, primary care notes, hospital admission notes, inpatient progress notes, pain management, mental health notes, social worker notes, and discharge summaries. To assess the impact of SBDH from both sources, we combined them to create 13 distinct SBDH factors (Appendix 2). In addition to individual-level SBDHs mentioned above, we also included neighborhood-level socioeconomic variable - area deprivation index (ADI)[3] which represents the socioeconomic status of a patient’s neighborhood. ADI includes state and national-level rankings of neighborhoods based on socioeconomic disadvantages. A higher ADI indicates a lower socioeconomic status. We linked each patient’s EHR data to the ADI database via their address zip code and discharge quarter of the calendar year to identify the corresponding national-level ranking and included that as a predictor.

We extracted all predictors from the observation window except diagnosis codes, SB and SBDH (excluding ADI). Diagnosis codes were extracted only from the discharge day as this yielded the best performance in our initial experiments. To capture prior documented SA and SI, we extracted SB data from any time before the current discharge date. Furthermore, we varied the time frame for SBDH to investigate how their proximity to discharge affects subsequent suicide. We chose 7 (a week), 30 (a month), 90 (3 months), 180 (6 months), 365 (1 year) and 730 days (2 years) as candidate time frames. To provide the model a sense of time-variability, we also used SBDH predictors extracted from all six time windows simultaneously.

In summary, we considered 619 candidate predictors (Table 1): 4 demographic variables, 283 diagnoses codes variables, 248 procedures codes variables, 50 medication codes variables, 2 SB variables, 6 structured SBDH variables, 12 NLP-extracted SBDH variables, 13 combined SBDH variables, and 1 ADI variable. Demographic and ADI variables were categorical, whereas the remaining predictors were constructed as binary variables - indicating the absence or presence.

### Predictor Screening

Predictor screening was performed on the binary features of diagnoses, procedure, and medication codes. First, we removed any of these predictors with a low prevalence of less than 1%. Next, for each remaining predictor, we fit a univariate logistic regression model of suicide death on the predictor and the demographic variables. We evaluated the p-values of the predictors from these univariate models and used the Benjamini-Hochberg procedure^27^ to control the false discovery rate (FDR) at 10%. Only predictors with an adjusted p-value smaller than 0.1 were used as candidate predictors to build the predictive models. Our two-stage screening reduced 87.4%, 78.51%, 75.77% and 71.41% of the predictors for the case-control cohorts with 7, 30, 90 and 180-day prediction windows respectively. Prior works suggest that predictor screening can help with noise reduction and substantially improve out-of-sample model performance^28,29^. We stress that SBDH variables were excluded from the screening stage as the focus of this work is on analyzing their impact on the prediction of suicide.

### Statistical Analyses

We employed three different machine learning (ML) methods for predictive modeling, namely, elastic-net logistic regression (ENL), random forest (RF), and multilayer perceptron (MLP). For the ENL and RF models, we used 10-fold cross-validation on the training data and performed grid searches over a wide range of hyperparameters to select the best models. For MLP, we used a 2-layer feed-forward network with ReLU^30^ as the activation function. To tune the hyperparameters of MLP, we set apart 20% of the training data as the validation set. As our cohorts had a case-control ratio of 1:5, we used cost-sensitive learning^31^ for all models to ensure that they prioritized suicide events as equally as non-suicide events. For ENL and RF, we averaged all metrics over the 10 folds. For MLP, we averaged the model performance over three runs with different seeds. We experimented with different combinations of predictors, as shown in Tables 2 and 3. For SBDH, we experimented with the following combinations: structured SBDH, NLP-extracted SBDH, combined SBDH, structured SBDH + ADI, NLP-extracted SBDH + ADI, and Combined SBDH + ADI.

To evaluate the models’ predictive performance on the test data, we examined various performance metrics on the test data, including the area under the receiver operator characteristic curve (ROC AUC), area under the precision recall curve (PR AUC), sensitivity, specificity, and positive predictive value (PPV). Since suicide is a rare event, we calculated sensitivity, specificity and PPV for different risk group sizes. A risk group size P for a predictive model indicates the fraction of the test set with the highest risk for suicide, as identified by the model. Following prior studies^22,32^ and our data statistics, we included 0.05, 0.10, 0.20 and 0.60 as different values for P. As this is a case-control study, we also reported adjusted PPV^33^. PPV denotes the probability of predicted high-risk patients with suicide death. The measurement of PPV is important as this indicates the chances of saving patients’ lives with interventions.

In addition, we conducted calibration analysis and measured predictor importance using the Kernel SHAP (Shapley Additive Explanations) method^34^. For each model, we chose PR AUC to select the best hyperparameter configuration. All analyses used Python 3.8, ENL and RF were implemented using scikit-learn^35^ 0.23.1 and MLP was implemented using PyTorch^36^ 1.5.1.

## Results

### Prevalence of Suicide

**Figure 1:**
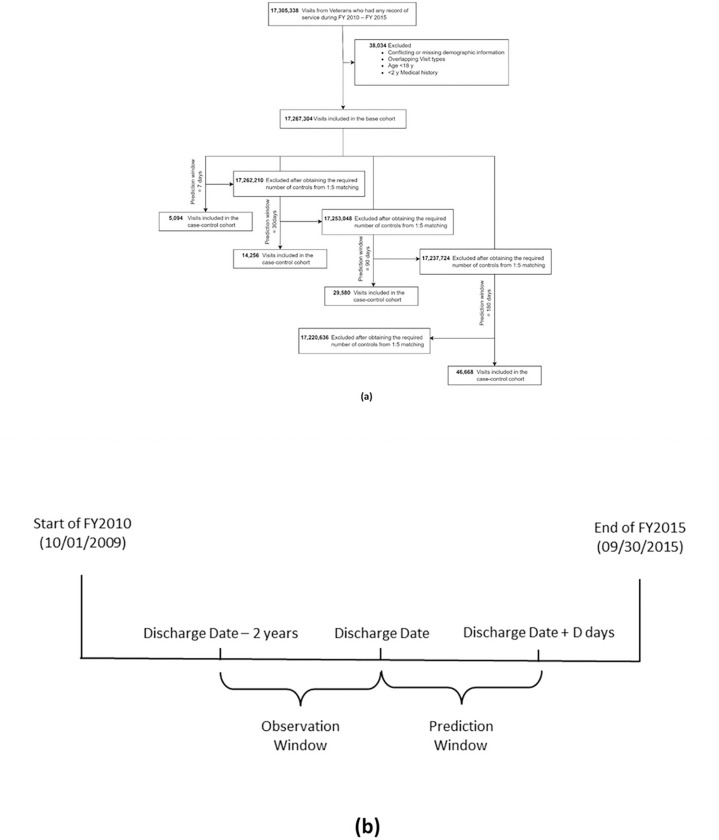
(a) Construction of the cohort and (b) our study timeline; D = {7, 30, 90, 180} (in days).

Out of 17,267,304 discharges from 2,987,006 Veterans (Fig. 1), 17,210,996 were eligible to be considered for the 7-day prediction with 849 cases, amounting to 0.005% suicide rate at the discharge level. At the patient level, the suicide rate within 7 days of discharge was 0.03%, with 849 suicide deaths from 2,703,173 patients. Similarly, the suicide rates within 180 days of discharge were 0.05% at the discharge level and 0.27% at the patient level. In summary, the 4 case-control cohorts for prediction windows 7, 30, 90 and 180 days consisted of 5,094 (849 cases and 4,245 controls), 14,256 (2,376 cases and 11,880 controls), 29,580 (4,930 cases and 24,650 controls) and 46,668 discharges (7,778 cases and 38,890 controls) respectively. More details are available in Appendix 3.

### Overall Model Performance

The results are shown in Tables 2 and 3. With ‘SBDH’ as predictors, we only reported results for the combinations that yielded the best PR AUC scores. We noticed incremental improvements across almost all models and prediction windows as we added a new predictor group. Adding codes and SB information always improved the AUC scores (Table 2). A similar trend can also be observed with SBDHs. However, the best SBDH setting for PR AUC did not always yield the best ROC AUC score.

ENL achieved the best AUC scores for the 7 and 30-day prediction windows, except MLP attaining the best PR AUC in the 7-day prediction window. In contrast, RF achieved the best AUCs across 90 and 180-day prediction windows. In general, models for the shortest prediction window (7 days) had the lowest ROC AUCs (74.44%–77.65%), and as prediction windows got longer, the models performed better with the highest ROC AUCs (77.39%–83.94%) obtained for the longest prediction window (180 days). PR AUC scores demonstrated a similar trend. AUC scores were almost always higher among outpatient ED discharges than inpatient discharges.

Across all prediction windows with the best predictor configuration, these models detected 12.98–24.58% of all deaths from suicide at the 5% risk tier (Table 3). This means that even considering only 5% of the discharges with the highest model-assigned suicide risk, a suicide intervention program based on these models can capture 12.98%–24.58% of patient discharges where the patients would otherwise die by suicide. Increasing the risk group size can help capture even more discharges, for example, 24.97%–41.14% at a 10% risk group size. PPVs and adjusted PPVs increase as the prediction window increases and the risk group size decreases. We obtained the highest adjusted PPV of 1.07% for the RF model over the 180-day prediction window at the 5% risk tier. This suggests that in the top 5% risk tier, patients from 1.07% of discharges would die by suicide within 180 days of their hospital discharges in the absence of any additional intervention program.

### Impact of NLP-extracted Predictors

In this study, we used an NLP system to extract SBDH from clinical notes. We compared our NLP-extracted SBDHs with structured and combined SBDHs. eTable5 lists all the SBDH combinations that yielded the best performance for each model at a specific prediction window. In half of the settings (6 out of 12), NLP-extracted SBDHs appeared as the best choice whereas structured SBDH performed better in four settings. We also found ADI to be helpful in most settings.

### Calibration and Predictor Importance

Out of the three models, RF is better calibrated than others (eFigure 1–2). However, there was no noticeable difference between a model with and without SBDH (eFigure 2). We also measured predictor importance using Kernel SHAP method (eFigure 4). Based on SHAP values, we identified predictors that pushed a model towards making positive predictions (suicide death) and predictors that did the opposite. We named them positive and negative predictors, respectively. Upon examining the top 30 positive predictors, we found that SA, SI, and the age group 79 or higher are the most common predictors across different models and prediction windows. In contrast, black race, female gender, and age 50–59 were the most consistent negative predictors in the top 30. Among diagnoses predictors, ‘Administrative/social admission’, ‘COPD’, ‘alcohol-related disorders’, and ‘anxiety disorders’ were the most common positive predictors. As for procedure categories, ‘anesthesia’ was a common positive predictor, whereas ‘cardiac stress tests’ was a common negative predictor. Among medications, ‘sedative hypnotics’ was a prominent positive predictor and ‘antidepressants’ was a common negative predictor. Among SBDHs, ‘Social isolation’ (NLP-extracted) and ‘violence’ (structured) were two of the most common positive predictors. We would like to emphasize that SHAP values do not indicate risk or protective factors; rather, they help rank predictors according to their usefulness for a task (suicide prediction) with respect to a model (ENL, RF, or MLP).

### Ensemble Learning

Ensembling is a popular technique for aggregating multiple models’ predictions to improve system robustness. Among various aggregator functions such as linear averaging, majority voting, boosting, etc., we chose linear averaging for our study. First, for each model, we averaged the prediction probabilities over all folds/runs and then, we averaged them over different models. We did this for the two best models (ENL and RF) and all three models. The results are shown in Table 4. We found that ensembling ENL and RF improved the AUC scores over the best single models for 7, 30, and 90-days prediction windows. However, the performance did not improve for the 180-days prediction window. Comparatively, ensembling all models was only helpful for prediction window 7. Overall, the RF model is still better calibrated than the ensembled systems (eFigure 3).

## Discussion

To the authors’ knowledge, this is the first case-control study to examine the roles of NLP-extracted SBDHs in predicting suicide among US Veterans. We found that models with SBDH predictors outperformed models without SBDH. For example, the RF model with no SBDH achieved an ROC AUC of 83.57% and a PR AUC of 57.38% in the 180-day prediction window. After adding ADI and NLP-extracted SBDH (timeframe = 730 days), the AUCs increased to 84.25% (0.81% improvement, 95% CI = 0.63–0.98, p-val < 0.001) and 59.87% (4.34% improvement, 95% CI = 3.86–4.82, p-val < 0.001) respectively. Moreover, when compared with structured SBDH, NLP-extracted SBDHs yielded competitive or better performance in most situations.

SBDH improved the performance for all cases, with ROC AUC improvements going up to 3.86% and PR AUC improvements up to 11.21%. This is consistent with prior studies^22,37^ where multiple SBDH factors were identified as important predictors for suicide after discharge from VA psychiatric hospitalization. However, they lacked a robust deep-learning-based SBDH extraction system from clinical notes. Our results also showed that all models benefitted from including NLP-extracted SBDHs in combination with other SDBHs or alone. This highlights the merit of harnessing clinical notes through NLP to enrich SBDH information for improved predictive modeling.

Our work showed that near-term prediction of suicide death is more challenging than longer-time predictions; as such, all models performed the best with 180-day prediction window, and the performance kept declining as the prediction window decreased. This may partly stem from the lack of adequate samples in shorter prediction windows, making it more challenging for any model to map the predictors to suicide. Other studies suggested that larger number of suicides over longer windows increase predictive models’ statistical power ^22,37^. They found that models built to predict suicide over longer windows outperform models built to predict over shorter windows when applied at those shorter windows.

We also ranked the predictors using their SHAP values (eFigure 4). We discovered that records of prior SA and SI are two of the most important predictors for death by suicide across all prediction windows. SA is well-established as a significant risk factor for suicide^3,38^. Data indicates that one out of every 100 attempt survivors dies from suicide within the first year, a risk approximately 100 times higher than that observed in the general population^39^. Furthermore, the risk of suicide can persist up to 32 years following an attempt^40^. A systematic review of 90 studies found a 6.7% suicide completion rate and a 23% non-fatal attempt rate^41^. We also found ‘social isolation’ and ‘violence’ as two of the most common positive SBDH predictors. Prior studies showed a higher association between social isolation and suicide risk^12,42–45^. Exposure to violence is also a well-known risk factor for suicidality^12,46,47^.

Using NLP to extract clinically relevant information from EHR notes is not new. Datta et al. reviewed 78 studies that utilized NLP to extract cancer-related information^48^. Mitra et al. developed a deep-learning-based NLP system to extract social determinants of health from EHR notes and showed their significant associations with suicide among US Veterans^12^. Bhanu et al. designed an NLP system to extract SB information from EHR notes^49^. Many other works also used NLP systems to detect suicidality in EHR notes^50–53^. However, ours is the first case-control study to incorporate NLP-extracted SBDHs as predictors for suicide death prediction.

Although predicting suicidal behavior has been an active area of research^17,22,28,54,55^, our study differs in the addition of NLP-extracted SBDH as predictors to analyze their impact on a diverse set of models’ predictive performance. Despite many existing studies on the prediction of suicide, integrating their findings to existing healthcare systems poses a multitude of challenges, such as lack of logistics support at the deployment centers, risk-benefit tradeoff, cost-effectiveness, a sense of false reassurance^22^, and generalizability, among others. Moreover, a systematic review of 17 suicide prediction studies found that all predictive models suffer from low PPV, regardless of the population distribution or risk tier ^56^, thus, making suicide prediction a challenging task. In contrast, Kessler et al. showed that predictive models have positive net benefit across plausible ranges of the PPV distribution^37^.

### Limitations and Future Work

Our study has several limitations. Firstly, the VA population’s demographic composition differs from that of the overall US population. Nonetheless, research utilizing VHA data has informed non-VA facilities in implementing enhanced clinical practices ^57–59^. Additionally, our study employed no VA-exclusive predictors, allowing for the extraction of the same predictors from EHRs at non-VA facilities for customized prediction models. Secondly, our analysis focused solely on outpatient emergency and inpatient care discharges. Expanding to include other hospital settings could enhance our comprehension of SBDHs’ impact on suicide. We leave this for our future work Thirdly, we restricted the observation window to 2 years to incorporate relatively current SBDHs but extending it to encompass historical SBDHs may enhance model predictions, a subject we will explore in future research. Lastly, we utilized the ADI, available only at the census tract block group level; however, we plan to investigate the recently proposed social vulnerability metric^60^ as an alternative in future studies.

## Conclusions

Ours is the first large-scale study to use NLP-extracted SBDH information from unstructured EHR data to predict suicide among Veterans. We showed that incorporating NLP-extracted SBDH exhibited improved predictive performance across different models and prediction windows. Consequently, integrating NLP-extracted SBDH into structured EHR data holds a promising avenue for the advancement of a more effective suicide prevention system.

## Figures and Tables

**Table 1:** List of predictors considered in our study.

Predictor Type	Description	Source
Demographic Variables	Race, gender, age, and marital status	Structured Data
SB Variables	SA and SI	Structured Data (Using VA CIPHER's phenotype algorithm)
Diagnoses Codes Variables	ICD-9 diagnoses codes grouped into 283 categories using CCS v2015	Structured Data (ICD-9 diagnoses codes)
Procedure Codes Variables	Procedure codes grouped into 248 categories using CCS v2015 and CCS v2022.1	Structured Data (ICD-9 procedure codes and CPT codes)
Medication Codes Variables	VANF classes grouped into 50 categories (Appendix 1)	Structured Data (VANF classes)
ADI	ADI database linked to the EHR data to obtain national-level ranking	Structured Data (Census/Survey)
Structured SBDH	6 SBDHs extracted from structured data	Structured Data (ICD-9 and VHA stop codes)
NLP-extracted SBDH	12 SBDHs extracted from 9 types of clinical notes using an NLP system^12^	Unstructured EHR notes

**Table 2:** Performance of different predictive models across different prediction windows. ROC AUC: area under the receiver operator characteristic curve; PR AUC: area under the precision recall curve; SD: standard deviation; Demo: demographic variables; SB: suicidal behaviors - attempt and ideation; Codes: diagnosis, procedure, and medication codes; SBDH: social and behavioral determinants of health; ENL: Elastic Net Logistic Regression; RF: Random Forest; MLP: Multilayer Perceptron.

Models	Predictor Configurations	ROC AUC (SD), %			PR AUC (SD), %		
		Outpatient ED	Inpatient	All Discharges	Outpatient ED	Inpatient	All Discharges
**Prediction Window = 7**							
ENL	Demo	50.32 (0.79)	48.66 (0.64)	61.34 (0.43)	16.64 (0.27)	17.29 (0.24)	22.22 (0.28)
	Demo + Codes	66.15 (0.60)	64.25 (0.42)	71.09 (0.45)	30.12 (1.00)	28.33 (0.87)	32.80 (0.71)
	Demo + Codes + SB	74.01 (0.48)	70.98 (0.31)	77.28 (0.27)	37.91 (0.92)	32.57 (0.89)	40.38 (0.57)
	Demo + Codes + SB + SBDH	74.63 (0.53)	70.66 (0.44)	77.65 (0.34)	39.11 (0.86)	32.54 (0.86)	41.50 (0.48)
RF	Demo	50.47 (1.01)	50.11 (0.84)	60.11 (0.59)	16.06 (0.25)	17.17 (0.70)	24.61 (0.76)
	Demo + Codes	65.93 (0.84)	60.97 (0.64)	71.46 (0.54)	26.84 (0.54)	24.36 (0.35)	30.16 (0.72)
	Demo + Codes + SB	72.68 (0.60)	69.00 (0.64)	76.51 (0.41)	32.00 (1.07)	30.73 (1.51)	34.89 (0.61)
	Demo + Codes + SB + SBDH	74.80 (0.84)	71.19 (0.79)	76.34 (0.68)	38.26 (1.60)	35.18 (1.93)	37.94 (0.82)
MLP	Demo	51.03 (0.93)	50.20 (1.56)	60.19 (0.97)	17.35 (0.44)	18.57 (0.43)	24.71 (1.31)
	Demo + Codes	63.16 (1.25)	60.48 (1.24)	69.54 (0.81)	27.93 (2.56)	26.19 (2.89)	33.15 (1.47)
	Demo + Codes + SB	69.42 (2.05)	66.43 (1.68)	74.21 (0.92)	31.85 (2.44)	29.07 (1.87)	41.12 (1.58)
	Demo + Codes + SB + SBDH	70.49 (1.08)	67.32 (1.03)	74.44 (0.77)	34.32 (1.10)	29.02 (0.85)	42.26 (1.26)
**Prediction Window = 30**							
ENL	Demo	58.20 (0.22)	56.35 (0.24)	62.96 (0.13)	21.49 (0.37)	19.48 (0.21)	24.32 (0.25)
	Demo + Codes	70.48 (0.19)	64.64 (0.18)	75.39 (0.15)	31.70 (0.25)	26.31 (0.21)	41.36 (0.33)
	Demo + Codes + SB	73.75 (0.15)	65.81 (0.17)	78.87 (0.15)	33.20 (0.17)	25.17 (0.29)	46.38 (0.21)
	Demo + Codes + SB + SBDH	73.73 (0.17)	65.82 (0.18)	78.87 (0.15)	33.74 (0.18)	25.08 (0.27)	46.90 (0.28)
RF	Demo	56.59 (0.29)	56.54 (0.76)	60.54 (0.16)	19.54 (0.32)	19.38 (0.63)	22.27 (0.20)
	Demo + Codes	72.05 (0.55)	65.26 (0.68)	75.54 (0.27)	31.05 (0.75)	26.54 (0.64)	36.32 (0.48)
	Demo + Codes + SB	74.58 (0.30)	67.45 (0.33)	78.49 (0.10)	31.56 (0.38)	26.81 (0.43)	40.46 (0.53)
	Demo + Codes + SB + SBDH	74.85 (0.31)	68.13 (0.23)	78.17 (0.22)	33.29 (0.52)	28.35 (0.41)	43.11 (0.67)
MLP	Demo	58.37 (0.87)	58.70 (1.29)	61.51 (0.66)	20.47 (0.57)	20.36 (0.38)	23.35 (0.67)
	Demo + Codes	65.15 (0.69)	61.73 (0.78)	66.43 (0.39)	26.92 (0.72)	23.77 (0.44)	29.95 (1.03)
	Demo + Codes + SB	67.74 (2.33)	62.35 (1.47)	70.84 (2.67)	27.56 (1.64)	23.06 (0.37)	35.12 (2.41)
	Demo + Codes + SB + SBDH	67.92 (1.12)	62.27 (0.55)	71.46 (1.33)	30.37 (1.52)	23.67 (0.20)	39.05 (1.68)
**Prediction Window = 90**							
ENL	Demo	60.73 (0.18)	54.99 (0.28)	64.17 (0.13)	22.11 (0.25)	17.82 (0.14)	24.98 (0.17)
	Demo + Codes	75.86 (0.15)	68.51 (0.18)	79.73 (0.11)	39.25 (0.21)	28.10 (0.27)	49.35 (0.11)
	Demo + Codes + SB	76.84 (0.15)	68.44 (0.22)	81.05 (0.12)	38.74 (0.21)	25.96 (0.20)	51.87 (0.14)
	Demo + Codes + SB + SBDH	77.09 (0.14)	69.40 (0.20)	80.94 (0.14)	39.86 (0.25)	26.55 (0.23)	52.73 (0.16)
RF	Demo	60.45 (0.37)	56.14 (0.84)	63.65 (0.23)	21.47 (0.34)	17.65 (0.43)	23.50 (0.31)
	Demo + Codes	77.49 (0.27)	69.58 (0.30)	81.05 (0.22)	39.01 (0.39)	26.69 (0.73)	51.01 (0.53)
	Demo + Codes + SB	78.62 (0.16)	69.82 (0.40)	82.51 (0.16)	38.61 (0.40)	25.67 (0.40)	52.34 (0.50)
	Demo + Codes + SB + SBDH	78.84 (0.32)	70.56 (0.41)	82.92 (0.26)	40.09 (0.38)	26.74 (0.70)	55.28 (0.41)
MLP	Demo	59.44 (0.11)	56.18 (0.13)	63.30 (0.13)	20.96 (0.34)	17.59 (0.09)	23.18 (0.84)
	Demo + Codes	71.49 (1.24)	65.19 (0.92)	74.39 (1.24)	34.54 (0.95)	24.07 (0.99)	41.25 (1.71)
	Demo + Codes + SB	70.05 (0.85)	63.08(1.19)	74.91 (0.51)	32.91 (1.33)	22.78 (1.08)	42.85 (0.99)
	Demo + Codes + SB + SBDH	73.16 (1.46)	66.34 (1.47)	77.61 (1.61)	35.27 (1.68)	24.97 (0.87)	46.38 (1.77)
**Prediction Window = 180**							
ENL	Demo	60.52 (0.21)	53.37 (0.47)	62.61 (0.12)	22.83 (0.51)	16.83 (0.23)	22.79 (0.17)
	Demo + Codes	77.99 (0.08)	69.74 (0.14)	80.81 (0.05)	43.05 (0.11)	27.00 (0.15)	51.19 (0.08)
	Demo + Codes + SB	78.95 (0.07)	70.63 (0.13)	81.70 (0.05)	43.41 (0.10)	26.39 (0.14)	52.58 (0.07)
	Demo + Codes + SB + SBDH	79.52 (0.08)	70.94 (0.15)	82.04 (0.05)	44.36 (0.11)	26.37 (0.14)	53.47 (0.09)
RF	Demo	62.81 (0.40)	55.00 (0.76)	63.98 (0.23)	23.84 (0.29)	17.21 (0.47)	22.98 (0.13)
	Demo + Codes	80.89 (0.30)	71.14 (0.45)	83.26 (0.25)	45.07 (0.77)	27.52 (0.74)	56.37 (0.49)
	Demo + Codes + SB	81.24 (0.27)	71.56 (0.29)	83.57 (0.21)	44.95 (0.66)	27.18 (0.57)	57.38 (0.31)
	Demo + Codes + SB + SBDH	81.70 (0.32)	71.81 (0.48)	83.94 (0.17)	47.07 (0.64)	27.55 (0.33)	59.95 (0.37)
MLP	Demo	61.40 (0.14)	51.84 (0.71)	63.52 (0.08)	23.33 (0.77)	15.86 (0.55)	23.17 (0.22)
	Demo + Codes	72.37 (1.97)	64.01 (1.76)	74.68 (1.86)	34.72 (0.69)	22.85 (0.52)	41.89 (1.68)
	Demo + Codes + SB	74.48 (0.11)	65.12 (0.58)	76.20 (0.30)	36.77 (0.17)	23.43 (0.56)	44.85 (0.33)
	Demo + Codes + SB + SBDH	74.72 (1.25)	65.59 (1.15)	77.39 (0.84)	37.25 (2.44)	23.44 (1.72)	46.53 (2.25)

**Table 3:** Performance of different predictive models across different prediction windows for the best predictor configuration. PPV: positive predictive value; ENL: Elastic Net Logistic Regression; RF: Random Forest; MLP: Multilayer Perceptron.

Models	Risk Group Size, P	Sensitivity (%)	Specificity (%)	PPV (%)	Adjusted PPV
**Prediction Window = 7**						
ENL	0.05	16.42 (0.58)	97.48 (0.12)	57.67 (2.03)	0.03 (0.00)
	0.10	29.27 (0.83)	94.06 (0.17)	50.80 (1.44)	0.02 (0.00)
	0.20	48.74 (1.85)	86.07 (0.39)	42.30 (1.61)	0.02 (0.00)
	0.60	88.68 (0.46)	46.03 (0.10)	25.60 (0.13)	0.01 (0.00)
RF	0.05	12.98 (1.03)	96.75 (0.22)	45.58 (3.63)	0.02 (0.00)
	0.10	24.97 (0.73)	93.16 (0.15)	43.33 (1.26)	0.02 (0.00)
	0.20	47.22 (1.62)	85.76 (0.34)	40.98 (1.41)	0.02 (0.00)
	0.60	88.41 (1.27)	45.98 (0.26)	25.53 (0.37)	0.01 (0.00)
MLP	0.05	15.89 (0.54)	97.36 (0.11)	55.81 (1.90)	0.03 (0.00)
	0.10	27.15 (1.43)	93.62 (0.30)	47.13 (2.48)	0.02 (0.00)
	0.20	48.57 (3.30)	86.04 (0.69)	42.15 (2.87)	0.02 (0.00)
	0.60	82.78 (1.87)	44.80 (0.39)	23.90 (0.54)	0.01 (0.00)
**Prediction Window = 30**					
ENL	0.05	18.59 (0.48)	97.83 (0.10)	64.00 (1.66)	0.12 (0.01)
	0.10	32.68 (0.57)	94.72 (0.12)	56.26 (0.98)	0.09 (0.00)
	0.20	53.86 (0.69)	87.04 (0.14)	46.37 (0.59)	0.06 (0.00)
	0.60	89.39 (0.32)	46.11 (0.07)	25.65 (0.09)	0.02 (0.00)
RF	0.05	15.28 (0.82)	97.14 (0.17)	52.61 (2.81)	0.07 (0.01)
	0.10	28.28 (0.79)	93.80 (0.16)	48.70 (1.36)	0.06 (0.00)
	0.20	51.52 (1.15)	86.55 (0.24)	44.35 (0.99)	0.05 (0.00)
	0.60	88.06 (0.87)	45.84 (0.18)	25.27 (0.25)	0.02 (0.00)
MLP	0.05	15.40 (0.74)	97.16 (0.15)	53.04 (2.56)	0.08 (0.01)
	0.10	26.09 (1.14)	93.35 (0.24)	44.93 (1.96)	0.05 (0.00)
	0.20	40.32 (1.02)	84.23 (0.21)	34.71 (0.88)	0.04 (0.00)
	0.60	79.88 (1.33)	44.14 (0.28)	22.92 (0.38)	0.02 (0.00)
**Prediction Window = 90**					
ENL	0.05	21.86 (0.16)	98.28 (0.03)	71.12 (0.53)	0.38 (0.01)
	0.10	38.19 (0.43)	95.48 (0.08)	62.13 (0.71)	0.25 (0.01)
	0.20	56.74 (0.50)	87.14 (0.10)	46.15 (0.41)	0.13 (0.00)
	0.60	90.25 (0.49)	45.89 (0.10)	24.47 (0.13)	0.05 (0.00)
RF	0.05	23.20 (0.28)	98.54 (0.05)	75.48 (0.91)	0.47 (0.02)
	0.10	38.46 (0.47)	95.53 (0.09)	62.56 (0.76)	0.26 (0.01)
	0.20	58.24 (0.48)	87.43 (0.09)	47.37 (0.39)	0.14 (0.00)
	0.60	92.31 (0.55)	46.29 (0.11)	25.03 (0.15)	0.05 (0.00)
MLP	0.05	23.20 (0.28)	98.54 (0.05)	75.48 (0.91)	0.47 (0.02)
	0.10	33.44 (0.97)	94.56 (0.19)	54.40 (1.58)	0.18 (0.01)
	0.20	51.38 (1.93)	86.10 (0.37)	41.79 (1.57)	0.11 (0.01)
	0.60	88.09 (1.57)	45.47 (0.31)	23.89 (0.43)	0.05 (0.00)
**Prediction Window = 180**					
ENL	0.05	23.20 (0.28)	98.54 (0.05)	75.48 (0.91)	0.47 (0.02)
	0.10	37.54 (0.22)	95.46 (0.04)	62.01 (0.36)	0.41 (0.01)
	0.20	56.31 (0.15)	87.19 (0.03)	46.45 (0.12)	0.22 (0.00)
	0.60	92.00 (0.21)	46.33 (0.04)	25.28 (0.06)	0.08 (0.00)
RF	0.05	23.20 (0.28)	98.54 (0.05)	75.48 (0.91)	0.47 (0.02)
	0.10	41.14 (0.41)	96.17 (0.08)	67.95 (0.67)	0.53 (0.02)
	0.20	60.86 (0.57)	88.09 (0.11)	50.21 (0.47)	0.25 (0.00)
	0.60	92.36 (0.58)	46.40 (0.11)	25.38 (0.16)	0.08 (0.00)
MLP	0.05	19.82 (1.44)	97.94 (0.28)	65.48 (4.74)	0.48 (0.10)
	0.10	33.29 (1.46)	94.62 (0.29)	54.99 (2.41)	0.31 (0.03)
	0.20	50.77 (1.06)	86.09 (0.21)	41.88 (0.87)	0.18 (0.01)
	0.60	87.61 (0.34)	45.46 (0.07)	24.07 (0.09)	0.08 (0.00)

**Table 4:** Performance of different predictive models, including ensembled systems, across different prediction windows. ROC AUC: area under the receiver operator characteristic curve, PR AUC: area under the precision recall curve.

Prediction Window	Models	ROC AUC, %	PR AUC, %
7	ENL	77.75	41.76
	RF	76.66	38.40
	MLP	75.62	42.88
	Ensemble (ENL + RF)	78.46	41.78
	Ensemble (ENL + RF + MLP)	78.39	43.69
30	ENL	78.96	46.94
	RF	78.50	43.48
	MLP	69.85	36.91
	Ensemble (ENL + RF)	79.91	48.51
	Ensemble (ENL + RF + MLP)	77.92	44.50
90	ENL	80.98	52.81
	RF	83.27	55.88
	MLP	79.36	48.54
	Ensemble (ENL + RF)	82.90	56.45
	Ensemble (ENL + RF + MLP)	82.94	55.53
180	ENL	82.08	53.52
	RF	84.36	60.67
	MLP	79.30	48.63
	Ensemble (ENL + RF)	84.23	58.75
	Ensemble (ENL + RF + MLP)	84.12	58.08

## Data Availability

The VHA EHR data are available under restricted access for Veterans’ privacy and data security laws, access can be obtained by relevant approvals through VA Informatics and Computing Infrastructure (VINCI) (contact: VINCI@va.gov). Individuals who wish to use this data for research purposes must fulfil the research credentialing requirements as outlined by the VA Office of Research and Development.

## References

[1] Suicide Data and Statistics | Suicide Prevention | CDC. https://www.cdc.gov/suicide/suicide-data-statistics.html.

[2] Wang H. Global, regional, and national life expectancy, all-cause mortality, and cause-specific mortality for 249 causes of death, 1980–2015: a systematic analysis for the Global Burden of Disease Study 2015. Lancet 388, 1459–1544 (2016). 27733281 10.1016/S0140-6736(16)31012-1 PMC5388903

[3] Suicide. https://www.who.int/news-room/fact-sheets/detail/suicide.

[4] National Veteran Suicide Prevention Annual Report. Office of Mental Health and Suicide Prevention (2021).

[5] Walby F. A., Myhre M. Ø. & Kildahl A. T. Contact With Mental Health Services Prior to Suicide: A Systematic Review and Meta-Analysis. Psychiatr Serv 69, 751–759 (2018). 29656710 10.1176/appi.ps.201700475

[6] Stene-Larsen K. & Reneflot A. Contact with primary and mental health care prior to suicide: A systematic review of the literature from 2000 to 2017. Scand J Public Health 47, 9–17 (2019). 29207932 10.1177/1403494817746274

[7] HealthyPeople.gov. Social Determinants of Health | Healthy People 2020. Healthy People 2020 Topics and Objectives 5–8 Preprint at https://www.healthypeople.gov/2020/topics-objectives/topic/social-determinants-of-health (2014).

[8] Blosnich J. R. Social Determinants and Military Veterans’ Suicide Ideation and Attempt: a Cross-sectional Analysis of Electronic Health Record Data. J Gen Intern Med 35, 1759–1767 (2020). 31745856 10.1007/s11606-019-05447-z PMC7280399

[9] Haw C., Hawton K., Gunnell D. & Platt S. Economic recession and suicidal behaviour: Possible mechanisms and ameliorating factors. International Journal of Social Psychiatry 61, 73–81 (2015). 24903684 10.1177/0020764014536545

[10] Kim H. M. Predictors of suicide in patient charts among patients with depression in the Veterans Health Administration health system: Importance of prescription drug and alcohol abuse. Journal of Clinical Psychiatry 73, (2012). 10.4088/JCP.12m07658 23140657

[11] Kaufman J. A., Salas-Hernández L. K., Komro K. A. & Livingston M. D. Effects of increased minimum wages by unemployment rate on suicide in the USA. J Epidemiol Community Health (1978) 74, 219–224 (2020). 10.1136/jech-2019-212981 PMC7549077 31911542

[12] Mitra A. Associations Between Natural Language Processing–Enriched Social Determinants of Health and Suicide Death Among US Veterans. JAMA Netw Open 6, e233079–e233079 (2023). 36920391 10.1001/jamanetworkopen.2023.3079 PMC10018322

[13] Kposowa A. J. Unemployment and suicide: A cohort analysis of social factors predicting suicide in the US National Longitudinal Mortality Study. Psychol Med 31, 127–138 (2001). 11200951 10.1017/s0033291799002925

[14] Nock M. K. Suicide and suicidal behavior. Epidemiologic Reviews vol. 30 133–154 Preprint at 10.1093/epirev/mxn002 (2008). 18653727 PMC2576496

[15] Dube S. R. Childhood abuse, household dysfunction, and the risk of attempted suicide throughout the life span: Findings from the adverse childhood experiences study. J Am Med Assoc 286, 3089–3096 (2001). 10.1001/jama.286.24.3089 11754674

[16] Dorr D. Identifying patients with significant problems related to social determinants of health with natural language processing. in Studies in Health Technology and Informatics vol. 264 1456–1457 (Stud Health Technol Inform, 2019). 31438179 10.3233/SHTI190482

[17] Kessler R. C., Bossarte R. M., Luedtke A., Zaslavsky A. M. & Zubizarreta J. R. Suicide prediction models: a critical review of recent research with recommendations for the way forward. Molecular Psychiatry 2019 25:1 25, 168–179 (2019). 10.1038/s41380-019-0531-0 PMC7489362 31570777

[18] Troister T., Links P. S. & Cutcliffe J. Review of predictors of suicide within 1 year of discharge from a psychiatric hospital. Curr Psychiatry Rep 10, 60–65 (2008). 18269896 10.1007/s11920-008-0011-8

[19] Jensen P. B., Jensen L. J. & Brunak S. Mining electronic health records: towards better research applications and clinical care. Nature Reviews Genetics 2012 13:6 13, 395–405 (2012). 10.1038/nrg3208 22549152

[20] Demner-Fushman D., Chapman W. W. & McDonald C. J. What can natural language processing do for clinical decision support? J Biomed Inform 42, 760–772 (2009). 19683066 10.1016/j.jbi.2009.08.007 PMC2757540

[21] U.S. Department of Veterans Affairs. National Center for Veterans Analysis and Statistics. Veterans Administration https://www.va.gov/vetdata/ (2016).

[22] Kessler R. C. Using Administrative Data to Predict Suicide After Psychiatric Hospitalization in the Veterans Health Administration System. Front Psychiatry 11, 390 (2020). 32435212 10.3389/fpsyt.2020.00390 PMC7219514

[23] Collins G. S., Reitsma J. B., Altman D. G. & Moons K. G. M. Transparent reporting of a multivariable prediction model for individual prognosis or diagnosis (TRIPOD): The TRIPOD statement. Ann Intern Med 162, 55–63 (2015). 25560714 10.7326/M14-0697

[24] Defense Manpower and Data Center, Sunnyvale, California. Joint, Department of Veterans Affairs (VA) and Department of Defense (DoD) Mortality Data Repository -National Death Index (NDI) Extract https://www.mirecc.va.gov/suicideprevention/Data/data_index.asp.

[25] US Department of Veteran Services. VA National Formulary - Pharmacy Benefits Management Services. https://www.pbm.va.gov/nationalformulary.asp.

[26] Liu Y. RoBERTa: A Robustly Optimized BERT Pretraining Approach. (2019).

[27] Benjamini Y. & Hochberg Y. Controlling the False Discovery Rate: A Practical and Powerful Approach to Multiple Testing. Journal of the Royal Statistical Society: Series B (Methodological) 57, 289–300 (1995).

[28] Su C. Machine learning for suicide risk prediction in children and adolescents with electronic health records. Translational Psychiatry 2020 10:1 10, 1–10 (2020). 10.1038/s41398-020-01100-0 PMC7693189 33243979

[29] Doshi R. P. Identifying risk factors for mortality among patients previously hospitalized for a suicide attempt. Scientific Reports 2020 10:1 10, 1–9 (2020). 10.1038/s41598-020-71320-3 PMC7495431 32938955

[30] Agarap A. F. Deep Learning using Rectified Linear Units (ReLU). (2018) doi:10.48550/arxiv.1803.08375.

[31] Elkan C. The Foundations of Cost-Sensitive Learning. International Joint Conference on Artificial Intelligence (2001).

[32] Martinez C. Deep sequential neural network models improve stratification of suicide attempt risk among US veterans. Journal of the American Medical Informatics Association (2023) doi:10.1093/JAMIA/OCAD167. PMC10746318 37769328

[33] Altman D. G. & Bland, j. M. Statistics Notes: Diagnostic tests 2: Predictive values. BMJ 309, 102 (1994). 8038641 10.1136/bmj.309.6947.102 PMC2540558

[34] Lundberg S. M. & Lee S. I. A unified approach to interpreting model predictions. in Advances in Neural Information Processing Systems vols 2017-Decem 4766–4775 (2017).

[35] Pedregosa F. Scikit-learn: Machine Learning in Python. Journal of Machine Learning Research 12, 2825–2830 (2012).

[36] Paszke A. PyTorch: An Imperative Style, High-Performance Deep Learning Library. Adv Neural Inf Process Syst 32, (2019).

[37] Kessler R. C. Evaluation of a Model to Target High-risk Psychiatric Inpatients for an Intensive Postdischarge Suicide Prevention Intervention. JAMA Psychiatry 80, 230–240 (2023). 36652267 10.1001/jamapsychiatry.2022.4634 PMC9857842

[38] Risk and Protective Factors | Suicide | CDC. https://www.cdc.gov/suicide/factors/index.html.

[39] Hawton K. & Heeringen K. Van. The International Handbook of Suicide and Attempted Suicide. (2000).

[40] Probert-Lindström S., Berge J., Westrin Å., Öjehagen A. & Skogman Pavulans K. Long-term risk factors for suicide in suicide attempters examined at a medical emergency in patient unit: results from a 32-year follow-up study. BMJ Open 10, e038794 (2020). 10.1136/bmjopen-2020-038794 PMC7783608 33130567

[41] Owens D., Horrocks J. & House A. Fatal and non-fatal repetition of self-harm: Systematic review. The British Journal of Psychiatry 181, 193–199 (2002). 12204922 10.1192/bjp.181.3.193

[42] Motillon-Toudic C. Social isolation and suicide risk: Literature review and perspectives. European Psychiatry 65, (2022). 10.1192/j.eurpsy.2022.2320 PMC9641655 36216777

[43] Cutright P. & Fernquist R. M. Marital status integration, psychological well-being, and suicide acceptability as predictors of marital status differentials in suicide rates. Soc Sci Res 34, 570–590 (2005).

[44] Stack S. Suicide: A 15-Year Review of the Sociological Literature Part II: Modernization and Social Integration Perspectives. Suicide Life Threat Behav 30, 163–176 (2000). 10888056

[45] Jang J. Risks of suicide among family members of suicide victims: A nationwide sample of South Korea. Front Psychiatry 13, (2022). 10.3389/fpsyt.2022.995834 PMC9614235 36311502

[46] MacIsaac M. B., Bugeja L. C. & Jelinek G. A. The association between exposure to interpersonal violence and suicide among women: a systematic review. Aust N Z J Public Health 41, 61–69 (2017). 27774704 10.1111/1753-6405.12594

[47] McLaughlin J., O’Carroll R. E. & O’Connor R. C. Intimate partner abuse and suicidality: A systematic review. Clin Psychol Rev 32, 677–689 (2012). 23017498 10.1016/j.cpr.2012.08.002

[48] Datta S., Bernstam E. V. & Roberts K. A frame semantic overview of NLP-based information extraction for cancer-related EHR notes. J Biomed Inform 100, 103301 (2019). 31589927 10.1016/j.jbi.2019.103301 PMC11771512

[49] Rawat B. P. S., Kovaly S., Pigeon W. R. & Yu H. ScAN: Suicide Attempt and Ideation Events Dataset. NAACL 2022 – 2022 Conference of the North American Chapter of the Association for Computational Linguistics: Human Language Technologies, Proceedings of the Conference 1029–1040 (2022) doi:10.18653/v1/2022.naacl-main.75. PMC9958515 36848299

[50] Cusick M. Using weak supervision and deep learning to classify clinical notes for identification of current suicidal ideation. J Psychiatr Res 136, 95–102 (2021). 33581461 10.1016/j.jpsychires.2021.01.052 PMC8009838

[51] Fernandes A. C. Identifying Suicide Ideation and Suicidal Attempts in a Psychiatric Clinical Research Database using Natural Language Processing. Scientific Reports 2018 8:1 8, 1–10 (2018). 10.1038/s41598-018-25773-2 PMC5943451 29743531

[52] Downs J. Detection of Suicidality in Adolescents with Autism Spectrum Disorders: Developing a Natural Language Processing Approach for Use in Electronic Health Records. AMIA Annual Symposium Proceedings 2017, 641 (2017). 29854129 PMC5977628

[53] Haerian K., Salmasian H. & Friedman C. Methods for Identifying Suicide or Suicidal Ideation in EHRs. AMIA Annual Symposium Proceedings 2012, 1244 (2012). 23304402 PMC3540459

[54] Kessler R. C. Predicting suicides after psychiatric hospitalization in US army soldiers: The Army Study to Assess Risk and Resilience in Servicemembers (Army STARRS). JAMA Psychiatry 72, 49–57 (2015). 25390793 10.1001/jamapsychiatry.2014.1754 PMC4286426

[55] De La Garza Á. G., Blanco C., Olfson M. & Wall M. M. Identification of Suicide Attempt Risk Factors in a National US Survey Using Machine Learning. JAMA Psychiatry 78, 398–406 (2021). 33404590 10.1001/jamapsychiatry.2020.4165 PMC7788508

[56] Belsher B. E. Prediction Models for Suicide Attempts and Deaths: A Systematic Review and Simulation. JAMA Psychiatry 76, 642–651 (2019). 30865249 10.1001/jamapsychiatry.2019.0174

[57] Oliver A. Public-sector health-care reforms that work? A case study of the US Veterans Health Administration. The Lancet 371, 1211–1213 (2008). 10.1016/S0140-6736(08)60528-0 18395583

[58] Hicken B. L. & Plowhead A. A model for home-based psychology from the veterans health administration. Prof Psychol Res Pr 41, 340–346 (2010).

[59] Fihn S. D. Insights From Advanced Analytics At The Veterans Health Administration. 10.1377/hlthaff.2014.0054 33, 1203–1211 (2017). 25006147

[60] Saulsberry L. The social vulnerability metric (SVM) as a new tool for public health. Health Serv Res 58, 873–881 (2023). 36401593 10.1111/1475-6773.14102 PMC10315381

